# Metastatic Renal Cell Carcinoma to the Parotid Gland in the Setting of Chronic Lymphocytic Leukemia

**DOI:** 10.1155/2012/265708

**Published:** 2012-02-19

**Authors:** Robert Deeb, Ziying Zhang, Tamer Ghanem

**Affiliations:** ^1^Department of Otolaryngology-Head and Neck Surgery, Henry Ford Health System, 2799 West Grand Boulevard, Detroit, MI 48202, USA; ^2^Department of Pathology, Henry Ford Health System, Detroit, MI 48202, USA

## Abstract

Renal cell carcinoma (RCC) is infamous for its unpredictable behavior and metastatic potential. We report a case of a patient with a complex history of multifocal renal cell carcinoma and chronic lymphocytic leukemia (CLL), who subsequently developed a parotid mass. Total parotidectomy revealed this mass to be an additional site of metastasis which had developed 19 years after his initial diagnosis of RCC.

## 1. Case Report

An 82-year-old male presented with a right preauricular mass for an 18-month duration. The mass was nontender and had grown rapidly in the previous 6 months. His past medical history is notable for chronic lymphocytic leukemia (CLL) diagnosed in 2004 which was not treated. Additionally, he has a history of right-sided RCC status post partial nephrectomy in 1990 as well as a tumor which developed in the left adrenal gland in 2007 for which he underwent total adrenalectomy and nephrectomy. Pathology from this excision was consistent with RCC, though it is unclear if this represented a metastatic lesion from his original site or a second primary tumor.

**Figure 1:**
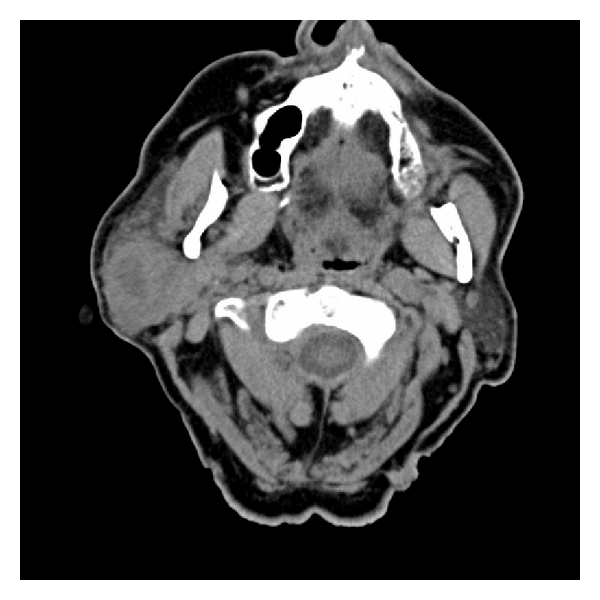
Preoperative CT scan.

CT scan of the neck revealed a 4 by 3.5 cm enhancing lesion in the right parotid gland involving the superficial and deep aspects of the gland (Figure 1). In order to differentiate between an intraparotid lymphoma and other etiologies of parotid tumors, fine needle aspiration (FNA) was performed. The specimen contained blood with increased amount of monotonous small-sized lymphocytes. A portion of the specimen was sent for flow cytometry analysis and result was consistent with chronic lymphocytic leukemia. Given the patient's medical history and the sanguineous nature of the needle aspirate, a broad differential diagnosis, including RCC, was still being considered.

**Figure 2:**
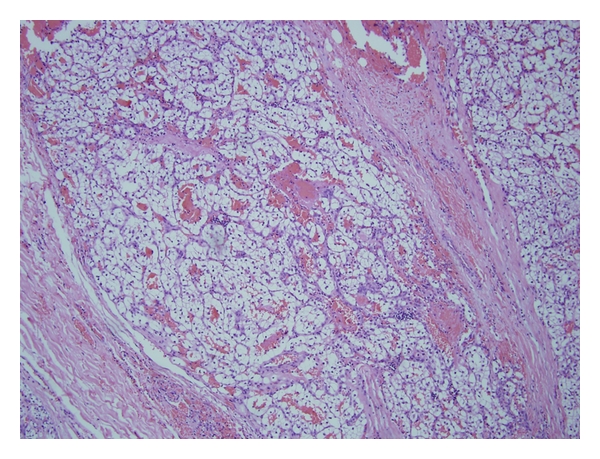
The tumor is characterized by sheets of round to polygonal cells interspersed with numerous thin-walled blood vessels (Hematoxylin-eosin, ×100).

 The patient underwent total parotidectomy. Once the lesion was encountered, frozen section analysis revealed metastatic renal cell carcinoma. The tumor encased the main trunk of the facial nerve and had a robust angiogenic response. Total parotidectomy was performed with facial nerve preservation. Permanent pathologic specimen revealed metastatic renal cell carcinoma, clear cell type (Figure 2). The patient was treated postoperatively with radiation to the parotid bed. Shortly after the radiation treatment the patient noted several lesions along the surgical bed. Biopsy performed of these lesions revealed RCC. He underwent a brief course of palliative chemo and is currently living comfortably with disease. No further intervention is planned.

## 2. Discussion

 Renal cell carcinoma (RCC) is infamous for its unpredictable behavior and metastatic potential. Although the most common sites for RCC metastasis are the lung, lymph nodes, bone, liver, adrenal, and brain, this neoplasm may involve any organ including the parotid as an unusual metastatic site [1, 2]. It is well described in the literature to metastasize to regions in the head and neck. In a case series of 65 patients with RCC presenting as metastatic lesion to the head and neck, 47 patients had cervical lymphadenopathy, and 18 patients presented at extranodal sites including the skin, thyroid, skull, pharynx, and lip, with no case in the parotid gland [3]. Moudouni et al. reported a 10-year lapse between the initial diagnoses of RCC and the metastasis to the submaxillary gland [4]. A recent review of all cases of RCC metastatic to the parotid gland published in the English language literature revealed a total of 25 such cases [5]. In 14 of these 25 cases, parotid metastasis was the initial presenting sign of malignancy in the kidney [5]. The time intervals from the removal of the kidney tumor to the appearance of the parotid mass ranged from several months to 10 years [5].

Though FNA is often utilized preoperatively in evaluating salivary gland malignancies, it is important to realize that there is a high false negative rate in evaluation of metastatic RCC to the parotid gland. Park and Hlivko reported a 33% incidence of false negatives or nondiagnostic results in their series [5]. Clear cell RCC is a challenging diagnosis for the pathologist as the differential diagnosis includes benign tumors, such as oncocytic hyperplasia, oncocytoma, myoepithelioma, pleomorphic adenoma, and sebaceous adenoma [6]. Immunohistochemical analysis is essential for making the diagnosis. Markers such as vimentin, CD-10, and EMA have been found to have particular utility in aiding to distinguish RCC from other neoplasms which may present in a similar manner.

The mechanism by which a renal cell carcinoma reaches the parotid gland is via hematogeneous spread. In fact, renal cell carcinomas are hypervascular tumors associated with multiple arteriovenous shunts due to release of VEGF and other angiogenic factors. Considering the fact that the kidneys receive 25% of circulating blood volume, renal cell carcinoma has a high hematogeneous spreading potential [7].

Another aspect of this case which warrants consideration is the fact that the patient had both RCC and CLL. Nishikubo et al. reported on a series of 8 patients in which they found an association between renal cell carcinoma and lymphoid malignancies [8]. In fact it was determined that the incidence of renal cell carcinoma and lymphoid malignancy occurring in the same patient is higher than expected in the general population by chance alone. Possible explanations for the relationship were hypothesized to be a genetic mutation common to both malignancies, environmental exposure, or an immunomodulatory effect of the first tumor causing a predisposition to the second.

The current case presented a difficult diagnostic as well as intraoperative challenge. First, establishing a diagnosis of RCC was not possible on FNA due to the vascular nature of the tumor. Given the patient's history of CLL, there was increased suspicion for intraparotid CLL. The treatment for this entity is chemotherapy instead of parotidectomy. Thus, when FNA was nondiagnostic, an open approach to the parotid was taken and frozen section analysis was performed to rule out lymphoma. The second aspect of this case which presented a challenge was the highly vascular nature of the tumor. Facial nerve preservation was still possible; however meticulous hemostasis was required during the dissection.

## 3. Conclusion

 Documentation of a case of metastatic renal cell carcinoma to the parotid gland is important to note as it serves to remind clinicians that any patient with a history of renal cell carcinoma is at risk for late metastases; including sites within the head and neck. Standard diagnostic measures such as FNA may not yield a definitive diagnosis and patients must be counseled accordingly. Thus, a high index of suspicion for metastatic disease should be given for patients with a history of renal cell carcinoma. Based on our literature review, this case represents the longest duration for recurrence of RCC metastatic to the parotid gland.
